# Insomnia is associated with suicidality in a sample of inpatients with psychiatric disorders

**DOI:** 10.1192/j.eurpsy.2026.11993

**Published:** 2026-07-31

**Authors:** M. Gojković, D. Mušić, M. Marković, Z. Pavlović, T. Cvetić, S. Pejović Nikolić, B. Savić

**Affiliations:** 1 Clinic for Psychiatry, University Clinical Centre of Serbia, Belgrade; 2 Department for psychiatry, General Hospital Novi Pazar, Novi Pazar; 3 School of Medicine, University of Belgrade, Belgrade, Serbia

## Abstract

**Introduction:**

Insomnia is a common yet overlooked symptom in psychiatric disorders, despite growing evidence showing its significant role in predicting symptom severity, illness progression and functional outcomes. Studies have consistently linked insomnia to an increased risk of suicidal ideation, planning, attempts, and completion. However, few studies examined this association in inpatients with psychiatric disorders.

**Objectives:**

The study aimed to investigate the prevalence of insomnia among adult patients admitted to inpatient psychiatric care across various diagnostic categories, and to explore its association with suicidality.

**Methods:**

A retrospective study was conducted using medical records from 1500 consecutive patients (mean age 46.4±15.1, female 61.9%) admitted to the Clinic of Psychiatry, University Clinical Center of Serbia, between June 2020 and June 2025. Demographic characteristics, psychiatric diagnoses, and history of substance use were collected. Presence of insomnia was defined as difficulty initiating and/or difficulty in maintaining sleep and/or early morning awakening using detailed psychiatric interview including mental status exam. Suicidality was defined as presence of suicidal ideation, plans or attempts at the admission. Logistic regression analysis examined the association of insomnia and suicidality controlling for age, gender, psychiatric diagnosis and history of substance use.

**Results:**

Prevalence of insomnia in the whole sample was 60.6%. Out of 232 patients with suicidality at admission, 78.2% had insomnia. Insomnia was significantly associated with increased risk of suicidality (OR=1.90, 95%CI=1.38-2.61, p≤.001) after adjusting for confounding factors.

**Conclusions:**

This study showed insomnia is highly prevalent at the time of psychiatric inpatient admission and is an independent risk factor for suicidality in psychiatric inpatients. Timely identification and management of insomnia are crucial in mitigating suicidal behavior and improving patient outcomes.

**Disclosure of Interest:**

None Declared

